# Supplementary material for: Dietary Patterns and Metabolic Disorders in Polish Adults with Multiple Sclerosis
Source: Nutrients. 2022 May 4;14(9):1927. doi: 10.3390/nu14091927 (PMC9104558; doi:10.3390/nu14091927)
Supplement: Supplementary file 1 [file nutrients-14-01927-s001.zip › nutrients-1705615-supplementary.pdf]

Table S1. Frequency of consumption of product groups (%).

Food group	Frequency consumption (% of the sample)					
	Never	1-3 times a month	Once a week	Few times a week	Once a day	Few times a day
Refined bread	7.9	12.7	9.1	25.2	27.3	17.9
Wholegrain bread	17.0	21.8	12.7	27.3	16.1	5.2
Refined groats, rice and pasta	5.5	19.7	34.8	37.6	2.1	0.3
Wholemeal groats, flakes and pasta	7.6	32.7	20.3	31.5	5.8	2.1
Fast food	25.8	64.5	6.7	3.0	0.0	0.0
Fried foods	7.3	25.2	28.8	34.8	2.7	1.2
Butter	11.5	13.0	6.1	23.9	27.9	17.6
Lard	72.4	16.4	3.9	5.8	1.2	0.3
Vegetable oils/margarine	32.1	14.5	12.4	27.9	8.8	4.2
Milk	20.9	14.8	11.5	18.2	23.9	10.6
Fermented milk beverages	9.4	20.3	19.4	37.3	12.7	0.9
Cottage cheese	12.4	28.8	23.0	30.0	5.5	0.3
Cheese	8.5	21.8	18.5	42.7	7.3	1.2
Cold cuts and sausages	12.4	13.9	14.2	39.7	15.8	3.9
Red meats	16.7	31.5	23.0	26.1	1.8	0.9
White meats	7.6	14.8	24.2	49.1	3.9	0.3
Fish	5.2	40.6	40.9	12.4	0.6	0.3
Eggs	2.7	14.8	30.9	46.1	5.2	0.3
Legumes	10.0	51.8	21.5	14.8	1.5	0.3
Potatoes	5.2	18.2	24.5	46.4	5.5	0.3
Fruit	0.9	2.1	8.2	34.8	30.3	23.6
Vegetables	0.3	2.1	3.3	39.4	23.9	30.9
Sweets	4.2	18.8	17.3	34.5	18.8	6.4
Instant soups	69.1	24.5	3.3	2.4	0.3	0.3
Canned meats	68.8	28.5	1.5	0.9	0.0	0.3
Canned vegetables	14.5	32.4	19.7	31.5	1.5	0.3
Juices	17.3	34.2	16.1	22.4	7.0	3.0
Vegetable juices	28.8	37.9	11.5	16.1	5.2	0.6
Sweetened drinks	43.9	36.1	7.9	9.4	1.5	1.2
Energy drinks	85.2	9.1	2.7	2.1	0.0	0.9
Alcohol	36.7	43.9	11.8	6.1	1.5	0.0

Table S2. Adherence to the DPs according to the diagnosed type of MS (%)

**Type of MS**	**Total N=330**	**Traditional Polish DP**			**Prudent DP**			**Fast food & convenience food DP**		
		T1 n=111	T2 n=107	T3 n=112	T1 n=110	T2 n=110	T3 n=110	T1 n=110	T2 n=109	T3 n=111
Relapsing-remitting MS (RRMS)	86.1	87.4	87.9	83.0	85.5	84.5	88.2	88.2	85.3	84.7
Secondary progressive MS (SPMS)	5.8	5.4	5.6	6.3	4.5	7.3	5.5	6.4	3.7	7.2
Primary progressive MS (PPMS)	8.2	7.2	6.5	10.7	10.0	8.2	6.4	5.5	11.0	8.1
p-value			ns			ns			ns	

DP – dietary pattern; MS – multiple sclerosis; T – tertile; ns – statistically insignificant difference.
